# Teasing Apart Translational and Transcriptional Components of Stochastic Variations in Eukaryotic Gene Expression

**DOI:** 10.1371/journal.pcbi.1002644

**Published:** 2012-08-30

**Authors:** Raheleh Salari, Damian Wojtowicz, Jie Zheng, David Levens, Yitzhak Pilpel, Teresa M. Przytycka

**Affiliations:** 1 National Center for Biotechnology Information, National Library of Medicine, Bethesda, Maryland, United States of America; 2 Institute of Informatics, University of Warsaw, Warsaw, Poland; 3 Bioinformatics Research Centre (BIRC), School of Computer Engineering, Nanyang Technological University, Singapore; 4 Laboratory of Pathology, National Cancer Institute, Bethesda, Maryland, United States of America; 5 Department of Molecular Genetics, Weizmann Institute of Science, Rehovot, Israel; MRC Laboratory of Molecular Biology, United Kingdom

## Abstract

The intrinsic stochasticity of gene expression leads to cell-to-cell variations, *noise*, in protein abundance. Several processes, including transcription, translation, and degradation of mRNA and proteins, can contribute to these variations. Recent single cell analyses of gene expression in yeast have uncovered a general trend where expression noise scales with protein abundance. This trend is consistent with a stochastic model of gene expression where mRNA copy number follows the random birth and death process. However, some deviations from this basic trend have also been observed, prompting questions about the contribution of gene-specific features to such deviations. For example, recent studies have pointed to the TATA box as a sequence feature that can influence expression noise by facilitating expression bursts. Transcription-originated noise can be potentially further amplified in translation. Therefore, we asked the question of to what extent sequence features known or postulated to accompany translation efficiency can also be associated with increase in noise strength and, on average, how such increase compares to the amplification associated with the TATA box. Untangling different components of expression noise is highly nontrivial, as they may be gene or gene-module specific. In particular, focusing on codon usage as one of the sequence features associated with efficient translation, we found that ribosomal genes display a different relationship between expression noise and codon usage as compared to other genes. Within nonribosomal genes we found that sequence high codon usage is correlated with increased noise relative to the average noise of proteins with the same abundance. Interestingly, by projecting the data on a theoretical model of gene expression, we found that the amplification of noise strength associated with codon usage is comparable to that of the TATA box, suggesting that the effect of translation on noise in eukaryotic gene expression might be more prominent than previously appreciated.

## Author Summary

The stochastic nature of gene expression leads to cell-to-cell differences in protein level referred to as *noise*. Expression noise can be disadvantageous, by affecting the precision of biological functions, but it may also be advantageous by enabling heterogeneous stress-response programs to environmental changes. Therefore various genes and gene groups might display various levels of expression noise. Importantly, gene expression is a multi-step process and the stochasticity of its individual steps, including transcription and translation, contributes to the resulting variability. Recent single cell analyses of gene expression in yeast have confirmed the theoretically predicted general trend where expression noise scales with protein abundance. However, some deviations from this basic trend have also been observed, prompting questions about the contribution of gene-specific features to such deviations. Accounting for noise heterogeneity in different gene groups, we revealed a clear relationship between noise and translation-related genomic features, specifically codon usage and 5′ UTR secondary structure. Our results suggest that the effect of translation on these deviations might be more prominent than previously appreciated, and provide important clues towards understanding expression stochasticity in yeast.

## Introduction

The stochastic nature of gene expression promotes cell-to-cell differences in protein level, usually referred to as *noise* [1], [2], [3]. Recent studies, both experimental and computational, have revealed that such cell-to-cell variability can be both disadvantageous [4], [5], [6], [7], [8], as variations in protein level might negatively affect the precision of signaling and regulation, and advantageous [9], [10], [11], [12], [13], [14], by enabling heterogeneous stress-response programs to environmental changes [10]. Expression noise has also been proposed to have an important impact on gene evolution [6], [15],[16]. These diverse roles are expected to be accompanied by complex and heterogeneous modes of noise regulation. In addition, feedback loops and other network motifs might be utilized to regulate noise [17], [18], [19], [20] or propagate it through regulatory networks [19], [21], [22], adding to the overall complexity.

The sources of variation in gene expression in an isogenic cell population are typically divided into two basic groups: (i) the intrinsic noise attributed to the inherent stochasticity of expression processes, and (ii) the extrinsic noise resulting from variation in cell state related to cell-cycle progression, cell size, subtle environmental differences, and other stochastic events that are external to the system – in this case external to the process of expression of an individual gene [1], [23], [24], [25], [26], [27], [28]. Several stochastic processes including transcription, translation, and mRNA and protein degradation can contribute to the intrinsic noise [2]. The relative contribution of these components is gene or gene-module specific. Basic factors can be gleaned from correlations between noise level and gene characteristics such as promoter structure, gene function, essentiality, chromatin density, and similar features [29]. In the context of the prokaryote *B. subtilis*, it has been observed that the predominant source of phenotypic noise strength is translational efficiency [30]. It has been proposed that in prokaryotes, low transcription but high translation rates produce protein bursts leading to strong fluctuations in the protein level [17], [30]. In contrast, noise in eukaryotic gene expression is assumed to be predominantly influenced by the dynamics of transcription [29], [31], [32], in particular transcription bursts [33], [34], [35]. Transcription bursts are not unique to eukaryotes and also have a clear impact on prokaryotic noise [36],[37]. Similarly, translation dynamics is expected to have an impact on eukaryotic gene expression. Along this line, Blake *et al.* demonstrated experimentally that codon usage can impact noise strength in eukaryotic gene expression and proposed that increased translational efficiency might have a substantial effect when coupled with a noisy transcriptional state [31]. Furthermore, a recent analysis of data collected by Bar-Even *et al*. showed some tendency for efficiently translated genes to have increased noise [38].

Single-cell analyses of gene expression in yeast provided an important step towards understanding noise etiology and demonstrated a general trend where expression noise scales with protein abundance [29]. This trend suggests that expression of most genes follows roughly a random stochastic process. Importantly, there are some deviations from this basic trend, indicating that gene specific factors might be altering this general behavior. Newman *et al*. measured these deviations with the DM measure, defined as the difference of the gene specific noise and the median noise for proteins with the same abundance, as estimated from the trend line for the relation between noise and abundance. We use the term *noise differential* to denote such deviation of the noise of an individual gene from the average trend, and thus DM is a measure of noise differential. Studies by Newman *et al*. [29] have uncovered a highly significant correlation of noise differential with several transcription regulation features, including the presence of a TATA box, but did not reveal such highly significant correlation with codon usage, the hallmark of translation efficiency [39], [40], [41]. However, untangling different components of expression noise is highly nontrivial. Intrinsic and extrinsic fluctuations can be separated experimentally by utilizing dual reporter measurements [1], [24], but experimental separation of transcriptional and translational components would additionally require single-cell measurements of both mRNA and protein copy numbers simultaneously [42].

To complement these studies, we used computational means to investigate the question of to what extent sequence features known or postulated to accompany translation efficiency can also be associated with noise differential. Specifically, we considered codon usage, as measured by the tRNA adaptation index (tAI), and 5′ UTR structure. High tAI is postulated to contribute to efficient translation elongation, while low secondary structure at the 5′ UTR has been shown to negatively correlate with ribosomal density [43], [44], [45], [46], [47], [48]. Thus, these two features may potentially correlate with amplification of the strength of transcription noise and noise differential.

We observed that ribosomal proteins display a different relationship between expression noise and codon usage as compared to other proteins. Focusing on nonribosomal proteins, we found that the above-mentioned features indeed have significant associations with noise differential. Among these features, the statistical significance of the association with tRNA adaptation index is the highest. We then used a theoretical noise expression model to decompose the protein abundance associated noise strength into two components: noise associated with transcription (represented by the presence of a TATA box) and noise putatively associated with translation (represented by high tAI), while controlling for the protein abundance. Strikingly, we found that the amplification of noise strength associated with high tRNA adaptation index is comparable to the amplification of noise strength associated with the presence of a TATA box. The noise factoring strategy that we introduced here for the purpose of uncovering relative interplay between these two factors is general and can be readily applied to tease apart other contributions of interest.

## Results

Noise is defined as the coefficient of variation 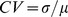, where 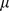 is the mean and 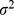 is the variance of experimental measurements. Recent single-cell studies of gene expression in yeast have uncovered a general trend where the squared coefficient of variation is inversely proportional to protein abundance [29], [32]. Supporting this understanding, Bar-Even *et al.* provided a theoretical argument for the hypothesis that expression noise results from a stochastic process where mRNA copy number follows the random birth and death process. Given this principal scaling property, attention has been turned towards uncovering systematic deviations from this abundance-related trend and correlating such deviations with specific gene features. Here we use the data gathered in the experiment of Newman *et al.*, where the trend line was only paralleling the Poissonian process for low to moderate expression levels. Using a two-dye experiment on a sample of highly expressed proteins, the experimenters demonstrated that these deviations from the Poissonian process for high expression levels are caused by extrinsic noise. Newman *et al.* accounted for this effect by introducing the DM measure (defined above).

### Heterogeneity of noise properties in different gene groups

**Figure 1:**
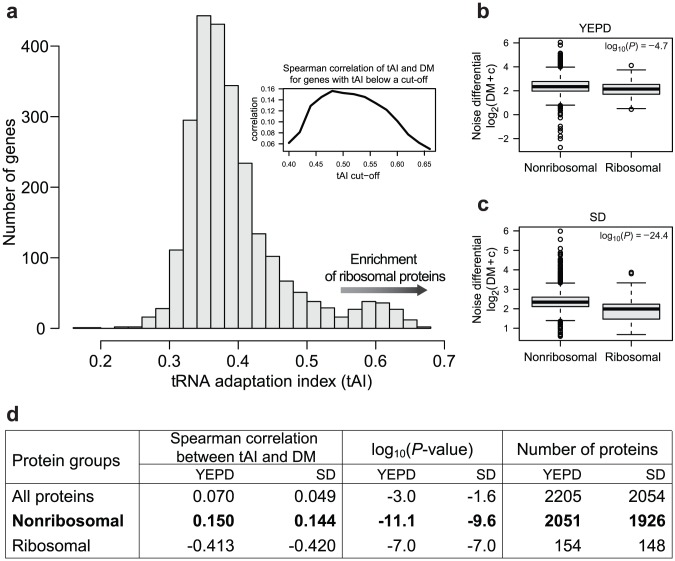
(**a**) Histograms of tRNA adaptation index (tAI) scores of budding yeast genes shows a long tail of high tAI values that is highly enriched in ribosomal genes (98 out of 153 genes with tAI>0.55, binomial test *p*-value<e-74). **Inset**: Spearman correlation between tAI and DM increases at a wide range of high tAI cut-off values. (**b,c**) Ribosomal and nonribosomal genes have different distributions of noise differentials (DM) in both YEPD and SD media – the ribosomal genes are significantly less noisy. For graphing purpose the DM values are shifted by constant c = 5 prior taking the logarithm. (**d**) Spearman correlation between tAI and noise differential (DM) for the whole dataset, including nonribosomal and ribosomal proteins in YEPD and SD media.

Given that translation efficiency has been found to impact cell-to-cell noise in prokaryotic organisms [30] and that translation efficiency has been demonstrated to have the potential to amplify transcription noise in eukaryotic cells [31], the low statistical significance of the correlation between codon usage and noise in Newman and colleagues' large-scale yeast study [29] was to some extent unexpected. Remarkably, we observed that the distribution of codon usage (as measured by tRNA adaptation index [49]) has a long tail (Figure 1a). Removing this tail at a wide range of cut-off values increases the significance of the Spearman correlation between tAI and DM (Figure 1a inset). We noticed that the genes at the tail of the tAI distribution are highly enriched in ribosomal genes – 98 out of 153 genes with tAI above 0.55 are ribosomal (binomial test, *p*-value<e-74). Comparing all ribosomal and nonribosomal genes, we found that these two groups have different distributions of noise differentials (DM) - the ribosomal group is significantly less noisy than the remaining genes (Figures 1b and 1c; Wilcoxon rank-sum test, *p*-values<e-4.7 and e-24.4 for YEPD and SD, respectively); the difference is also statistically significant for equal sample sizes (Supplementary Table S1). In addition, the correlation between DM and tAI in ribosomal group was negative (Figure 1d; Spearman correlation −0.4, *p*-values<e-7 for both YEPD and SD). In contrast, we observed a highly significant positive correlation between noise differential and tRNA adaptation index in the group of nonribosomal genes, suggesting a robust contribution of the translation process to expression noise in this group of genes (Figure 1d; Spearman correlation, *p*-values<e-11.1 and e-9.6 for YEPD and SD, respectively). Given this different relation between noise differential and codon usage for these two groups of genes, we removed all ribosomal genes (see Materials and Methods) from further analyses and focused only on nonribosomal genes.

**Figure 2:**
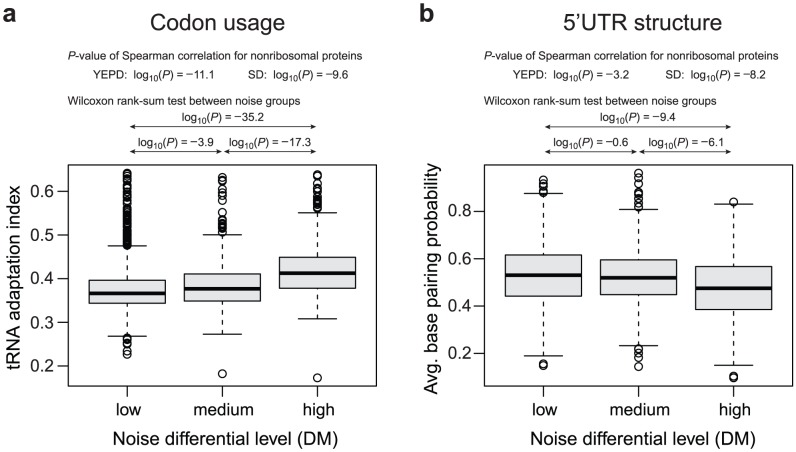
Comparison of translation related mRNA features: (**a**) codon usage (tRNA adaptation index and (**b**) average base pairing probability at the 5′ UTR mRNA structure for different noise differential levels. The nonribosomal genes were subdivided into three groups: low, medium and high noise genes, according to their noise differential levels in YEPD and SD media.

Additionally, we combined the measurements from both growth media and subdivided the nonribosomal genes into three groups according to noise level: low, medium and high noise genes (see Materials and Methods for precise description of the grouping). Figure 2a shows statistically significant differences in tRNA adaptation index between these three noise differential levels (Wilcoxon rank-sum test, all *p*-values<e-3.9).

### Variations in mRNA secondary structure correlate with variations in noise differential

The structure of messenger RNA is known to affect translation efficiency [43], [44], [45], [46], [47], [48]. A low level of secondary structure at the 5′ UTR of mRNA correlates with increased ribosomal density, leading to an increased translation rate [43], [44], [45], [46], [47], [48], [50], [51]. More importantly, simultaneous translation of several protein molecules from the same mRNA molecule leads to a deviation from the Poissonian model of gene expression (see also Fraser et al. [4]). Therefore, we conjectured that the level of secondary structure at the 5′ UTR of mRNA might impact expression noise. To test this hypothesis we computed, for each gene in the nonribosomal group and each position within the gene, the gene base pairing probability in RNA structure (Materials and Methods). Indeed, we observed statistically significant inverse correlation between 5′ UTR secondary structure and noise differential (Figure 2b; Spearman correlation, *p*-values<e-3.2 and e-8.2 for YEPD and SD, respectively). Using the same subdivision of the genes into three groups as for the codon usage above, we observed that the noisiest genes were characterized by the lowest base pairing probability in the 5′ UTR region, while the least noisy genes had the most structured 5′ UTR. The differences between the highly noisy group and remaining two groups are also statistically significant (Figure 2b; Wilcoxon rank-sum test, *p*-values<e-9.4 and e-6.1 for comparison with low and medium noise genes, respectively; Supplementary Table S1 for equal size set sampling).

Potentially, if a gene is optimized for rapid but noisy expression then optimization of codon usage is likely to be accompanied by other efficiency boosting features such as unstructured 5′ UTR. Indeed, there is a highly statistically significant inverse correlation between 5′ UTR structure and tAI (Spearman correlation −0.2; *p*-value<e-22). Partial correlation analysis indicates that these correlations remain statistically significant when controlling for the third factor (Supplementary Table S2). The correlation of 5′ UTR with DM in YEPD was the weakest and after controlling for tAI or TATA was only marginally significant (*p*-value = 0.0366 and *p*-value = 0.0134 respectively). Therefore we did not include 5′ UTR in our next analysis, where we used a theoretical noise expression model to decompose the gene-specific amplification of noise strength into a transcriptional component attributed to the TATA box and a putative translational component attributed to tAI.

### Decomposing noise strength amplification into TATA and tAI associated components

Given the above-demonstrated relationship between the sequence features associated with translational efficiency and noise differential, we wanted to see whether we could capture the interplay between transcriptional and translational features in a more quantitative way. Theoretical models imply that both transcription and translation bursts lead to an increase in noise strength [17], [20], [26], [52], [53] – equivalent to the Fano factor as defined by 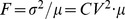 (note that for a Poissonian process 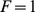, while a process with 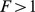, i.e. 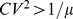, is considered to be noisy). Specifically, if 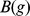 is the transcription burst size of gene 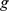 and 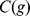 is the translation burst size then, ignoring any other noise contributors, noise strength can be approximated as the product 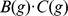 (see Materials and Methods for additional derivation). Here, we would like to capture the relative contributions of the 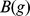 and 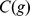 components to the noise strength.

We focused on the tAI measure of codon usage as a translation-related feature that correlated most strongly with noise differential in our study. The correlation with 5′ UTR RNA structure was considerably weaker, so we did not consider it in this analysis. Defining noise strength amplification defined as a fold-increase of noise strength for constant protein abundance, we wanted to capture the interplay between the noise strength amplification that can be attributed to increased tAI and the noise strength amplification that can be attributed to presence of a TATA box. The latter relation was identified in previous studies as one of the most prominent transcriptional noise contributors [26], [54], [55]. We note that noise strength amplification (a concept similar to the noise residual [32]) implies an increase of noise differential. This is explained more naturally in terms of theoretical models (see Materials and Methods).

**Figure 3:**
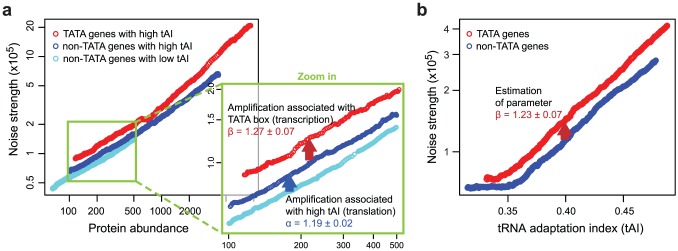
Decomposing noise strength amplification into TATA and tAI associated components. (**a**) The trend lines for the relation between protein abundance and noise strength (YEPD medium) in three groups of genes: TATA genes with high tAI (red), non-TATA genes with high tAI (blue) and non-TATA genes with low tAI (cyan). High and low tAI mean upper and lower tertile of tAI distribution, respectively. The abundance region where all three trend lines overlap is enlarged. The shift between TATA and non-TATA genes, both with high tAI, represents an amplification associated with the TATA box (transcription feature), β = 1.27±0.07, while the shift between non-TATA genes with high and low tAI represents an amplification associated with high codon usage (translation feature), α = 1.19±0.02. (**b**) The trend for the noise strength (YEPD medium) as a function of codon usage efficiency (tAI) for TATA genes (red) and non-TATA genes (blue). The shift between these two trend lines provides an alternative estimate of 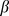, representing the impact of the TATA box on noise strength.

To estimate noise strength amplification due to TATA box presence and compare it to noise strength amplification attributed to high tAI, we devised two complementary approaches. First, we divided all nonribosomal genes into three groups: TATA genes with high tAI, non-TATA genes with high tAI, and non-TATA genes with low tAI (Figure 3a). All three groups overlap on a certain abundance interval (contain a subgroup of proteins with similar abundance), allowing us to estimate noise strength amplification. Specifically, the average ratio of Fano factor values for TATA genes with high tAI to those for non-TATA genes with high tAI provides an estimate of the noise strength amplification that can be associated with the TATA box to be 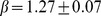 (YEPD). Along the same lines, the average ratio of Fano factors for non-TATA genes with high tAI to those for non-TATA genes with low tAI provides an estimate 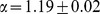 (YEPD) of noise strength amplification that can be associated to high tAI values. The estimates based on data in SD medium are similar (Supplementary Table S3). This indicates that, on average, the noise strength amplification that accompanies high tAI values is comparable to the noise strength amplification that accompanies the presence of a TATA box.

As an alternative approach to estimate 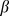, we divided the genes into two groups: genes containing a TATA box and genes without the TATA box. We looked at noise strength as a function of codon usage (Figure 3b) and directly compared noise strength for TATA and non-TATA genes at the same codon usage values. If the TATA box leads to noise strength amplification 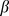, then the trend for the relation between codon usage and noise strength for TATA genes should be related to the trend for non-TATA genes by a multiplicative factor 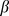. This is indeed what we observed (Figure 3b). Assuming that TATA and non-TATA genes with the same tAI are, on average, under the influence of the same extrinsic noise, then this graph provides an estimate of 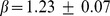 (YEPD), well within the error bars of the previous estimation. This consistency, and the fact that the noise strength amplification observed in Figure 3b is consistent over the full range of codon usage, including low codon usage genes that are typically not very abundant, suggests that this estimate is not affected by extrinsic noise.

## Discussion

We examined the deviations from the general trend where expression noise scales with protein abundance. In principle, all processes involved in gene expression can contribute to such a deviation. However, pinpointing the relative contributions of these components is nontrivial. Our study clearly demonstrated a relation between genomic features associated with the translational process and intrinsic noise. High codon usage and low content of 5′ UTR secondary structure correlated with increased expression noise differential. The relative contribution of transcription and translation features to noise varied for different gene groups. In particular, high codon usage ribosomal genes are characterized by low noise differential. This heterogeneity is probably a primary reason behind the difficulties in uncovering the interplay between various contributors.

We also performed an initial estimation of the noise strength amplification associated with TATA box presence and noise strength amplification that accompanies genes with high codon usage. Surprisingly, on average, the amplification of noise strength that accompanies high codon usage is comparable to the amplification that accompanies TATA box.

While precise factoring of intrinsic noise into its constitutive components requires additional experimental data, the approach developed in this study allowed us to provide initial estimates of the relative contributions of transcriptional and translational noise factors. The noise factoring strategy that we introduced in this study is general and can be used to measure the relative impact of other noise factors.

## Materials and Methods

### Data sources

#### Ribosomal genes

The list of ribosomal genes was downloaded from the website of the SGD (Saccharomyces Genome Database) project under the GO term “Structural constituent of ribosome” (GO: 0003735, http://www.yeastgenome.org/cgi-bin/GO/goTerm.pl?goid=3735). It consists of 224 manually curated genes.

#### TATA –genes

The list of genes with TATA-containing and TATA-less promoters was obtained from a study by Basehoar *et al.* [56].

#### Noise and groups

We used single-cell profiling measurements of protein abundance and its cell-to-cell variations in *S. cerevisiae*, provided by Newman *et al.* [29]. The experimenters measured steady-state protein levels for cells grown in rich (YEPD) and minimal (SD) media. The *DM* values (referred here as *noise differentials*) were used to quantify gene-specific noise levels.

In this study, we classified genes according to noise level in both YEPD and SD media. We subdivided the genes into three groups using somewhat arbitrary thresholds for noise level (DM):

- high noise genes - genes with high noise level in at least one medium, i.e. DM_SD_>4 or DM_YEPD_>4;
- medium noise genes - genes with medium noise level in at least one medium but without high noise level in any medium, i.e. 1<DM_SD_≤4 or 1<DM_YEPD_≤4 and DM_SD_≤4; DM_YEPD_≤4;
- low noise genes - genes with low noise level in any medium where it was measured but without medium or high noise level in any medium, i.e. DM_SD_≤1 and DM_YEPD_≤1.

This classification resulted in 1432 genes in the low level, 571 genes in the medium level, and 315 genes in the high level group (total 2328 genes). Note that all ribosomal genes were removed from this analysis.

### Computing codon usage

We measured the translation efficiency by the tAI score, which takes into account the availability of tRNA for each codon, and the efficiency of the codon-anticodon coupling. We followed the definition of Tuller *et al.* [57].

### Computing base pair probability

All *S. cerevisiae* gene sequences with 100 bases upstream were downloaded from the UCSC genome browser [58] (June 2008 genome assembly of *S. cerevisiae*). We used the RNAplfold program from the Vienna RNA package [59] to compute the base pair probabilities of RNA secondary structure for all sequences in our dataset. RNAplfold computes local pair probabilities; the probabilities are averaged over all windows of given size L that contain the base pair. We ran RNAplfold with a few different window sizes in the range of 100–300 nucleotides and obtained quite similar results. The results presented here are based on a window size of 150 nucleotides. The composite pairing probability profile was built by averaging base pairing probabilities over all genes for each position in the profile.

### Noise model

Assuming that protein abundance is characterized by two parameters – the mean number of protein production bursts per cell cycle, and the mean number of proteins produced per burst – Friedman *et al.* established the correspondence between these parameters and steady-state distribution. In a system where both transcription bursts and translation bursts are assumed to contribute to the total burst in protein abundance, noise strength 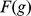, can be decomposed further into transcriptional and translational components. Specifically, if 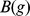 is the transcription burst size of gene 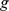 and 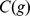 is the number of proteins translated from one mRNA molecule then, ignoring any other noise contributors, the noise strength 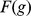 can be approximated as 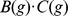.

As an alternative derivation, following Raser *et al.* [26] (equation [6] SOM), we simplified the expression for noise strength as
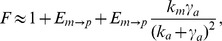
where 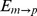 is the average number of proteins produced from a single mRNA molecule, *k_α_* is the promoter activation rate, *k_m_* is the RNA production rate, and γ_α_ is the promoter closing rate. Assuming that the protein production rate is proportional to codon usage 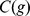 and that the transcription-related noise strength is attributed to a transcription burst size 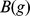 we have
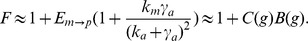



### Noise trends and computing noise strength amplification

We used a trend line to smooth out fluctuations in the noise data and to show an underlying pattern more clearly. To compute the trend line, we used the moving average method with overlapping windows of fixed number of genes. We used two different window sizes depending on the size of the gene group: 100 and 300 genes for data in Figure 3a and 3b, respectively. To estimate noise strength amplification (parameters α and β), we divided the interval where trend lines of considered gene groups overlap into bins, and for each bin we computed the ratio of mean trend values between each pair of gene groups. As an estimate of each parameter we took the average value of computed ratios.

### Computational platforms

All calculations and statistical analyses were performed using the R statistical environment (http://www.r-project.org). Scripts were written in the Python programming language (http://www.python.org/).

## Supporting Information

Table S1 *P*-values for Wilxocon tests performed on the original data groups and on sampled groups.
(XLSX)

Table S2 Pairwise Spearman's rank correlation between DM, tAI and 5′ UTR structure for nonribosomal genes, and partial correlations controlling for tAI, 5' UTR and TATA presence.
(XLSX)

Table S3 Estimates of noise strength amplification associated with the TATA box (parameter α) and the tRNA adaptation index (parameter β), based on data from YEPD and SD media.
(XLSX)
