# Correction: Teasing Apart Translational and Transcriptional Components of Stochastic Variations in Eukaryotic Gene Expression

**DOI:** 10.1371/annotation/4498943d-525d-4ac1-8375-ae78e67ea0c8

**Published:** 2012-10-08

**Authors:** Raheleh Salari, Damian Wojtowicz, Jie Zheng, David Levens, Yitzhak Pilpel, Teresa M. Przytycka

The copyright statement for this article is incorrect. The correct copyright statement should read: “This is an open-access article, free of all copyright, and may be freely reproduced, distributed, transmitted, modified, built upon, or otherwise used by anyone for any lawful purpose. The work is made available under the Creative Commons CC0 public domain dedication.” The copyright statement in the PDF version of this article is correct.

